# Study of the Integration of the CNU-TS-1 Mobile Tunnel Monitoring System

**DOI:** 10.3390/s18020420

**Published:** 2018-02-01

**Authors:** Liming Du, Ruofei Zhong, Haili Sun, Qiang Zhu, Zhen Zhang

**Affiliations:** 1 Beijing Advanced Innovation Center for Imaging Technology, Capital Normal University, Beijing 100048, China; limingado@163.com (L.D.); z770867381@163.com (Q.Z.); zzrango@163.com (Z.Z.); 2 College of Resource Environment and Tourism, Capital Normal University, Beijing 100048, China

**Keywords:** mobile tunnel monitoring system, three-dimensional laser scanning, multi-sensors, cross-section, deformation detection, dynamic measurement

## Abstract

A rapid, precise and automated means for the regular inspection and maintenance of a large number of tunnels is needed. Based on the depth study of the tunnel monitoring method, the CNU-TS-1 mobile tunnel monitoring system (TS1) is developed and presented. It can efficiently obtain the cross-sections that are orthogonal to the tunnel in a dynamic way, and the control measurements that depend on design data are eliminated. By using odometers to locate the cross-sections and correcting the data based on longitudinal joints of tunnel segment lining, the cost of the system has been significantly reduced, and the interval between adjacent cross-sections can reach 1–2 cm when pushed to collect data at a normal walking speed. Meanwhile, the relative deformation of tunnel can be analyzed by selecting cross-sections from original data. Through the measurement of the actual tunnel, the applicability of the system for tunnel deformation detection is verified, and the system is shown to be 15 times more efficient than that of the total station. The simulation experiment of the tunnel deformation indicates that the measurement accuracy of TS1 for cross-sections is 1.1 mm. Compared with the traditional method, TS1 improves the efficiency as well as increases the density of the obtained points.

## 1. Introduction

With the rapid development of China’s construction industry, the construction of railways and urban rail transit infrastructure have also increased at an unprecedented rate. A large number of high speed railways with high tunnel line ratios have been appearing, and the proportion of subway to urban rail transit is up to 80%. The number and length of tunnels increase annually, and thus, the monitoring and detection of the state of the tunnel structure, maintenance and disease control of tunnels are becoming particularly important. In the future, there will be a large number of tunnels that need to be detected and maintained. The safety status of the tunnel geometry directly affects the usability of the tunnel. Untimely maintenance of a tunnel not only wastes money but also affects the normal use of the tunnel, shortens the service life of the tunnel, and sometimes even endangers the safety of drivers and pedestrians. High speed railways and subway tunnels have long operation times and short skylight periods, and thus, there is a special need for a rapid, accurate and automatic detection method for the tunnel. Presently, the means of tunnel deformation detection mainly include the total station, a laser tunnel cross-section profiler, terrestrial laser scanners, etc. The total station detects the tunnel by measuring the characteristic points of level diameter or obtaining discrete cross-sectional points. Based on a small number of cross-sectional points, the tunnel cross-section lines can be fitted to express the displacement and deformation of the tunnel. The measurement accuracy of the total station is high, but the low measurement efficiency and low density of cross-sectional points means that it cannot express the security situation of the inner wall of a tunnel in detail [1]. The laser cross-section profiler needs to set out points on the route’s central line each time, in order to ensure the measuring head is perpendicular to the central line; its efficiency is also very low [2]. The three-dimensional laser scanning technology can quickly obtain the three-dimensional point cloud data of the inner wall of a tunnel. It has high efficiency and can obtain high density points, but the terrestrial three-dimensional laser scanner still requires multiple stations to be set up, especially when the tunnel is long; in addition, all the stations must be stitched together, and algorithms must be used to extract the cross-sections later, which often takes a long time because of the large amount of data involved [3,4].

In contrast, the mobile three-dimensional laser scanning system is more suitable for the scanning of a tunnel due to its high efficiency and accuracy. In the mobile three-dimensional laser scanning system, the differential global positioning system (DGPS) and inertial measurement unit (IMU) are often used as the integrated navigation module, and when combined with imaging sensors, they can acquire three-dimensional point clouds [5,6,7]. However, the tunnel is a closed space, lacks global positioning system (GPS) signals and has poor lighting, and many traditional mobile laser scanning systems are unsuitable for this special environment. Therefore, scholars at home and abroad have carried out studies on the three-dimensional laser scanning system of tunnels, which generally can optimally integrate various sensors and measurement instruments to directly obtain the tunnel cross-section for analysis. Mobile tunnel scanning systems have already been developed at home and abroad, most of which are measured in a tunnel with a three-dimensional laser scanner or a cross-section profiler on a track inspection trolley. These include the GPR3000 track inspection trolley with a cross-section profiler100 and the GPR5000 track inspection trolley with a Leica HDS4500 laser scanner, which were researched and developed by the Amberg Company in Switzerland [8]. These systems integrate a displacement sensor, clinometer sensor, odometer and prism in addition to the measurement device and can obtain the tunnel point cloud with absolute coordinates. In addition, the SiTrack One mobile track scanning system researched and developed by Leica in Switzerland can obtain the tunnel point cloud data with a relative accuracy of 3–5 mm by integrating a three-dimensional laser scanner, an odometer, IMU, GPS antenna, and target sphere [9]. To obtain the position and posture of the system in the tunnel environment, the sensors integrated in these systems all contain an IMU and odometer, however, the expensive IMU and odometer will lead to the high cost of the system. Therefore, the applicability of these systems has yet to be perfected and has not been a good promotion in the domestic market. Chai et al. [10] combined the helical mode of the Faro Focus3D scanner with the angle encoder and obtained the three-dimensional point cloud of a tunnel. The clearance inspection of the tunnel can be completed using this system, but its applicability to tunnel deformation detection has not been studied thoroughly. Wang [11] used the subway train as the carrier of the scanner and completed the deformation detection of cross-sections, but the accuracy of this method is greatly influenced by the speed of the vehicle.

The research of the tunnel deformation detection method by scholars at home and abroad mainly focuses on the method of tunnel cross-section extraction, which is based on point cloud data obtained by a terrestrial laser scanner. When the cross-sectional points are extracted from the point cloud data of the tunnel, the circular or elliptical model can be used to fit the cross-sectional lines to analyze the tunnel deformation [12,13,14,15]. The research on the algorithm of cross-section extraction mainly focuses on how to make the cross-section orthogonal to the tunnel more accurately, which is mainly divided into the following categories:

(1) Rotate the point clouds of the tunnel to make them parallel to a certain coordinate axis and then cut the cross-section by the coordinate axis [16].
(2) Project the three-dimensional point clouds to the horizontal plane and convert them into a two-value image. The image is used to extract the central axis of the tunnel from the horizontal plane, and then the later rigorous correction algorithms are adopted to obtain the cross-sections [17].
(3) The point clouds of the tunnel are projected onto two planes. Then, the spatial central axis of the tunnel is extracted by the fine mathematical method and used to cut the cross-sections [18].
(4) The cross-sections are extracted by the design spatial central axis of the tunnel directly [19].

Even though the cross-sections that are orthogonal to the tunnel can be obtained by the point cloud processing algorithm, there is an inevitable error accumulation in the calculation process, which affects the extraction accuracy of the cross-section and further affects the accuracy of the deformation analysis.

In this study, the CUN-TS-1 mobile tunnel monitoring system is developed and presented based on deep research of monitoring methods of tunnels. Considering that the maintenance of tunnels in the operation stage mostly rely on the ring number or certain mileage of the tunnel wall to locate the deformation, and the absolute coordinate position is not very useful, the system locates the cross-sections through the odometers, and then corrects the mileages through the longitudinal joints of the tunnel segment lining, which significantly reduces the cost of the system. In the process of tunnel data collection, the system detects the tunnel by the human hand push method, with no need to be located by a control measurement, and can efficiently obtain the three-dimensional cross-sectional point clouds directly by setting the laser scanner into the helical mode. Meanwhile, the original cross-sections obtained by the system are all orthogonal to the tunnel due to the rigid structure of the trolley body, which eliminates the later algorithm for the adjustment of the posture of cross-sections. Furthermore, to set up the parameters of sensors in the data acquisition process, control multi-sensors to start working at the same time and integrate the multi-sensors data, a data acquisition software matched with the system is developed. In addition, the system can analyze the deformation of arbitrarily selected cross-sections by the developed data processing software. In this paper, the hardware integration, software development and data processing methods of the CUN-TS-1 mobile tunnel monitoring system are discussed, and the accuracy of the system is verified through the measurement of the actual tunnel.

## 2. System Integration

### 2.1. Hardware Integration Scheme

The CNU-TS-1 mobile tunnel monitoring system integrates a three-dimensional laser scanner, displacement sensor, two odometers, notebook computer and power supply on the tunnel monitoring trolley.

The type of the laser scanner is Faro Focus3D 120. Its scanning accuracy can reach ±2 mm when the scanning distance is 25 m, and the measurement rate is up to 976,000 points per second. The horizontal scanning angle range and vertical scanning angle range of the laser scanner is 0°–360° and 0°–305°, respectively. During the mobile measurement process, the scanner is set into a helical mode to scan the tunnel. In this type of operation mode, the scanner operates in 2D profiling mode, where the rotating mirror axis is used, while the horizontal axis stays locked, and a scan line obtained by the laser scanner can be considered to be a cross-section. Finally, the three-dimensional coordinates and intensities of cross-sectional points are obtained, in which the y coordinates along the direction of the tunnel are zero. The realization of the function of the odometer is mainly through the ZSP3806-003 incremental photoelectric shaft encoder with a maximum mechanical speed of 6000 rpm. In the process of measurement, the distance can be calculated by the radius of the wheel and the number of cycles passing through the wheel; then the data are transmitted directly to the computer through the serial port. The TS1 installed an odometer on two wheels on the left and right sides of the tunnel monitoring trolley to improve the accuracy of the mileage data. In addition, the GH type MTS displacement sensor of the MTS System Company is used to measure the gauge. The sensor uses non-contact technology to monitor the displacement of an active magnet, which is not easily affected by the monitoring environment. The accuracy of the nominal repetition measurement of the displacement sensor is up to ±0.0025 mm, the nonlinearity is ±0.05 mm, and the measurement resolution can reach up to 0.005 mm. Because the body of the tunnel monitoring trolley has a “T” structure, the track data on one side can be obtained during the data acquisition process, and the track data on the other side can be calculated by combining the obtained tack data with the gauges measured by the displacement sensor. These track point clouds can be used for a later clearance inspection of the tunnel. The notebook computer is the control terminal and data processing terminal of the laser scanner, odometer and displacement sensor. It controls the sensors by software and realizes the data acquisition, processing and output.

**Figure 1:**
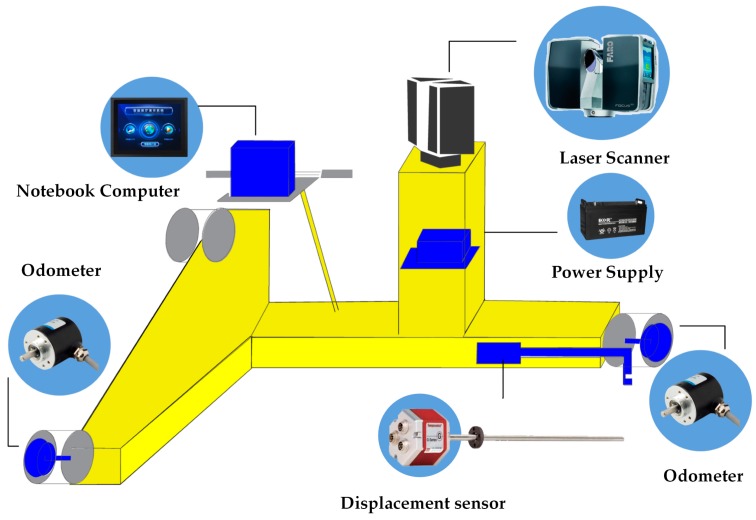
Hardware Integration Scheme of the TS1.

The hardware integration framework of the TS1 is shown in Figure 1. The posture of the trolley body and tunnel has a rigid change because of the rigid structures of the hardware and scanner, so the cross-sections collected by the tunnel monitoring trolley are all orthogonal to the tunnel. The system is suitable for the a shield tunnel with a standard gauge of 1435 mm, and the measurement processes are carried out in a human push method.

**Figure 2:**
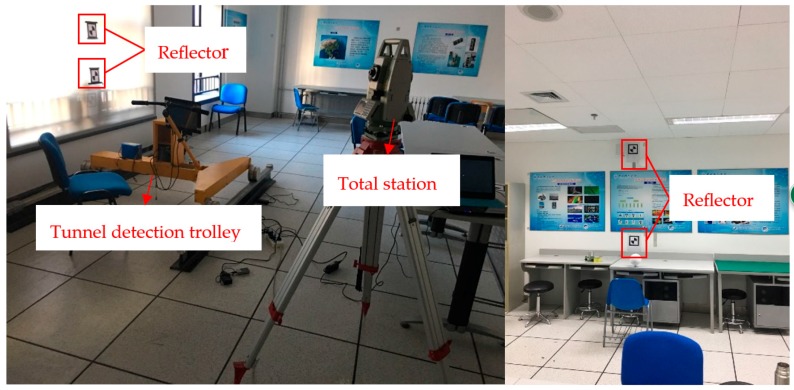
Sensor calibration of TS1.

After the hardware system is integrated, the time and space of the system must be synchronized [20]. Considering that data transmission will lead to inaccuracies in the time, the timing of the multi-sensors is unified to the timing of the central control computer system, which interpolates the data obtained by the odometers and displacement sensor based on the number of cross-sections that are received by computer in the unit time. Then, the time synchronization of the scanner, odometers and displacement sensor is realized. The main purpose of space synchronization is to establish the spatial correspondence of the scanner coordinate system, trolley body coordinate system and track coordinate system, which involves the obtaining of the calibration parameters of the monitoring system. The calibration process has to be carried out on the track which geometric parameters have already been obtained. First, the spatial relationship between the trolley body coordinate system and scanner coordinate system is established by using the total station coordinate system as the transition coordinate system. Then, the transformation relationship between the scanner coordinate system and the track coordinate system is obtained by combining the posture of the trolley body and the attitude of the track. The initial calibration experiment can be carried out in a laboratory environment, which is shown in Figure 2. Repeated disassembly and assembly of the equipment may lead to tiny changes of the equipment calibration parameters; however, because the trolley body is a rigid structure, and the relative position of the trolley body and track can be approximated as invariable. Therefore, the onsite calibration can be completed by confirming the relationship between the scanner and the trolley body.

### 2.2. Software Design and Implementation

The software of the laser scanner cannot control the data acquisition process of the tunnel monitoring trolley and cannot realize the later data processing and result analysis. On the basis of integration of the tunnel monitoring equipment, the data acquisition software and data processing software have been developed in this study.

**Figure 3:**
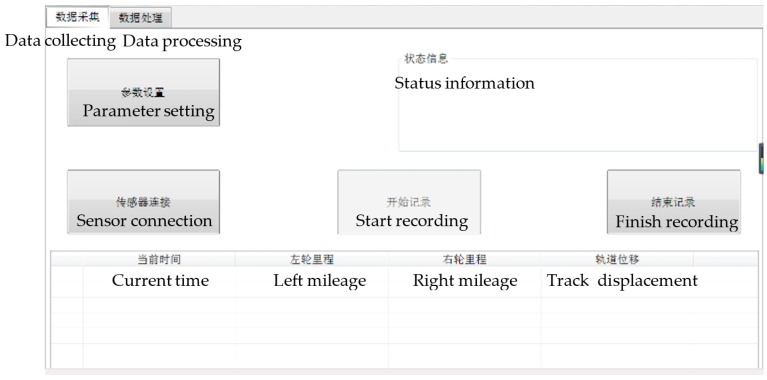
Interface of the data acquisition software.

To control the multi-sensors of the system to collect data and fuse the multi-source data, the data acquisition software is developed on the platform of Visual Studio 2010 C++ (Microsoft, Redmond WA, USA). The software consists of two modules, which are data collecting and data processing; its main interface is shown in Figure 3. The data collecting module controls the data acquisition process of the tunnel monitoring trolley by setting relevant parameters of each sensor, such as the serial ports, current mileage, IP address, storage path and file name. After the parameter setting is completed, each sensor is established a connection and the tunnel monitoring trolley can be carried out from the mileage of the starting point to the mileage of the end point. Finally, the data collected by each sensor are automatically recorded in their corresponding pre-set folder in the software. Furthermore, to ensure that the error in the later data fusion process is small enough, the uniform walking speed should be ensured in the process of data acquisition. The experiment shows that according to a normal human walking speed, a 1–2 cm interval of adjacent cross-sections can be achieved in the direction of the tunnel, and the density of cross-sectional points in every section can reach 2–3 mm.

The main function of the data processing module is to fuse the multi-sensors data, and the principle used is to align the data of each sensor based on the time stamp. All acquired data should be first checked for continuous linear change. If there is a significant error point, it should be eliminated as a gross error; otherwise, the next step should be done. Finally, the average values of the mileage data obtained by the two odometers are calculated and considered to be the final mileage data. As mentioned in the previous part of the article, the y coordinates of all point clouds obtained through the helical mode of the laser scanner are zero; thus, the mileage data are used as the y coordinates (along the tunnel direction) of the point clouds. The software finally outputs point cloud data in LAS format, which includes x and z coordinates obtained by laser scanner, mileages, gauges and intensities of the cross-sectional point clouds. The original y coordinates of point clouds obtained by laser scanner are eliminated in the LAS format, and the x coordinates, mileages and z coordinates are considered as the new three dimension coordinates of the cross-sectional points.

Because of the accuracy limit of the odometer, the errors of the mileages accumulates gradually with the advancement of the acquisition process. Therefore, the point cloud data need to be corrected after the data fusion process is completed to improve the positional accuracy of each cross-section. The correction process is done mainly using the longitudinal joints between the tunnel segments. First, the original tunnel point clouds are projected to the plane through the isometric tangent projection, and the tunnel inner wall image is generated using the reflectance information of the data to complete the extraction and location of longitudinal joints in the tunnel. Because the width of the design tunnel segment is a certain value, the process of correction can be completed according to the width of tunnel segment, the number of longitudinal joints in the tunnel and the mileages of the starting point and end point. Meanwhile, the tunnel point clouds of the direct and reverse measurements can be registered together after the mileage correction is completed.

**Figure 4:**
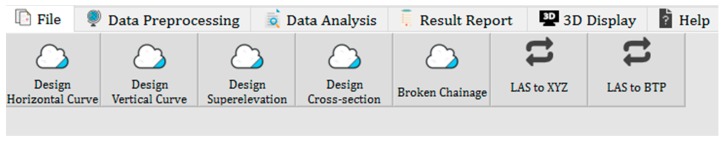
Interface of the data processing software.

Because of the large amount of point cloud data and because the time available for data acquisition in the tunnel is very limited, the analysis of the cross-section cannot be completed in real time. Therefore, in this paper, the tunnel data processing software is specially developed based on the QT platform. The basic processing of point cloud data (including denoising, scanning line generation, tunnel extraction, track extraction, and central axis generation, etc.) can be completed with the software. Concurrently, the cross-sections of arbitrary mileages or arbitrary mileage intervals can be selected for analysis, and the analysis results can be exported in the form of reports. In addition, the three-dimensional modelling and display of tunnel can be completed using the software, and the main interface of the data processing software is shown in Figure 4.

## 3. Methods

After system integration is completed, its accuracy must be verified to provide service for later production. For a tunnel at an operational stage, the detection of deformation usually takes into account the relative deformation of a tunnel segment at a certain ring or a mileage position. In this paper, the accuracy of the system is verified by analyzing the deformation of the tunnel using a variety of methods.

### 3.1. Accuracy Verification Method of TS1

The presence of ancillary facilities (cables, power distribution boxes, lighting equipment, etc.) in the tunnel result in the presence of noises in the tunnel cross-sectional points; thus, it is necessary to remove unused points [21,22]. An iterative elliptical fitting method is used here to filter the noises in the cross-sectional points [23]. In the filtering method, according to the shortest distances from the cross-sectional points to the fitted ellipse, the mean and standard deviation can be calculated. When difference of the distance from a cross-sectional point to the ellipse and the mean value is three times greater than the standard deviation, the point is considered to be a noise and removed; meanwhile, the de-noised cross-sectional points of the first iteration are used to fit the cross-sectional line in the second iteration until all the noises are filtered. In the iteration process, the RANSAC [24] algorithm is used to estimate the parameters of ellipse fitting.

#### 3.1.1. External Coincidence Accuracy Verification Method

Convergent deformation is often used as an important index to measure the deformation of a tunnel in a traditional cross-section analysis [16,17,18,19]. Measuring the tunnel convergent diameter can effectively monitor and record the roundness and deformation of the tunnel and obtain the dynamic change and trend of the tunnel cross-section over time. In this paper, the least squares method [25] and the circle model are used to fit the de-noised cross-sectional points, and the diameter of the circle is regarded as the convergent diameter of the cross-section. The convergent diameter deviation of the cross-sections measured by the TS1 and total station at the same position is considered to be the external coincidence accuracy of the TS1.

#### 3.1.2. Internal Coincidence Accuracy Verification Method

**Figure 5:**
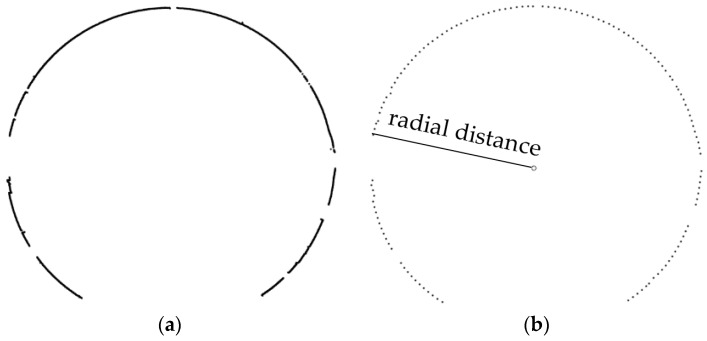
Resampling of cross-sectional points. (**a**) Before resampling; (**b**) after resampling.

In addition, in order to verify the stability of the TS1 for deformation detection, the point deformation value of each cross-sectional point can be calculated based on the point cloud data, and then the absolute mean deformation value (the mean value of the absolute value of all point deformation values in each section) of each cross-section can be calculated and considered to be the whole deformation of the cross-section. The whole deformation deviation of the cross-sections measured by direct and reverse measurement at the same mileage can be considered to be the internal coincidence accuracy of TS1. To calculate the deformation of each cross-sectional point at every angle interval more accurately, the cross-sectional points obtained by the TS1 should be resampled first because of their high density. The resampling method we used here is to select the nearest cross-sectional point approaching to the direction line at each angle interval and the selected cross-sectional points are used to calculate the deformations in the corresponding direction. Figure 5a,b is the results before resampling and were resampled at an interval of 2° from the cross-section.

The intersection of the cross-section and the design central axis of the tunnel is the design center of the cross-section [26]. In the previous literature, the distance between the measured cross-sectional point and the design center of the cross-section is often used as the radial distance, and then the point deformation of the tunnel is measured by comparing the radial distance to the design radius of the tunnel segment [27]. This method requires that the point cloud data have absolute coordinates and very high registration accuracy; otherwise, the registration errors will accumulate to the calculation of the later deformation quantity. Our system can be used to analyze the relative deformation of the tunnel directly by using the measured original cross-sections. To make a contrast, the circle center fitted by the cross-sectional points which are obtained through direct measurements can be considered as the reference center and instead of the design center of the cross-section. The radial distance of each cross-sectional point collected by direct and reverse measurements can be obtained by calculating the distance from the cross-sectional point to the reference center. By comparing the radial distances with the radius of design tunnel segment, the deformations of all the cross-sectional points at various angles can be obtained. Then, the whole deformation deviation of each cross-section can be calculated.

### 3.2. Calculation Method of Cross-Section Mileage Based on Absolute Coordinates

To compare the accuracy of the TS1 with the total station, the cross-sections at the same position should be selected from the two sets of data obtained by TS1 and total station, respectively. As discussed in the previous part of the article, the TS1 uses mileage to locate the cross-section and obtains three-dimensional coordinates of point clouds in the vehicle body coordinate system, but the total station obtains the absolute coordinates of the cross-sectional points directly. The coordinates of the two sets of points from the TS1 and the total station refer to different systems; therefore, they cannot be used together unless a reference system transformation is performed. Since the density of the cross-sections obtained by the TS1 can reach 1–2 cm, but the cross-sections measured by the total station are sparse (a cross-section is usually measured at intervals of multiple tunnel segments in actual tunnel detection). For comparison, we first calculate the mileage of each cross-section measured by the total station, and then the cross-sections that have the same mileages as the total station are selected from the cross-sectional point clouds obtained by the TS1.

There are three types of horizontal curve lines, which include the straight line, transition curve line and circular curve line [28]. All types of curves vary linearly with the length of the arc, and the curve unit can be used to represent any type of curve line; the mileage of any point out of the curve unit can be calculated by a unified mathematical model which is expressed the corresponding relationship between the surface point and the curve unit [29]. Therefore, the mileage of each cross-section can be calculated by combining the absolute coordinates of the instrument station point of cross-section measured by the total station and the design horizontal curve line data of the tunnel.

**Figure 6:**
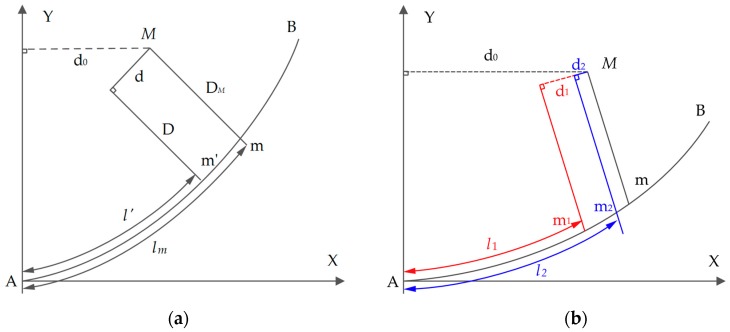
Mileage calculation of a point outside the curve. (**a**) The principle of the algorithm; (**b**) the iterative process.

As shown in Figure 6a, *AB* is route central line of the tunnel, *M* is an instrument station point of an arbitrarily cross-section measured by the total station, and *m* is the projection point of point *M* in the route coordinate system. Because the coordinates and mileage of the starting point *A* and the end point *B* in the route coordinate system can be calculated by the design data of the route, the mileage of point *M* in route *AB* can be obtained by calculating the arc length $l_{m}$ of the point *m*. If an arbitrary point $m^{\prime }$ whose arc length is known on *AB* can be selected, its arc length is $l^{\prime }$. According to Equation (1), the coordinates ($X_{m^{\prime }}$, $Y_{m^{\prime }}$) of point $m^{\prime }$ in the route coordinate system can be calculated, and then the shortest distance d from point *M* to the normal line D of point $m^{\prime }$ can be solved according to Equation (2). If d approaches 0, that is, when $m^{\prime }$ and *m* overlap, the mileage of point *M* can be calculated by the arc length $l^{\prime }$. Therefore, the solution can be obtained using the principle of infinite approach and the iterative method, that is, make point $m^{\prime }$ close to point *m* infinitely through continuous iteration.

As shown in Figure 6b, in the first iterative process, the shortest distance $d_{0}$ from point *M* to the normal line of starting point *A* is considered to be the initial arc length $l_{1}$, and the shortest distance $d_{1}$ from point *M* to the normal line of point $m_{1}$ can be calculated by Equations (1) and (2). If $d_{1}$ is greater than the threshold, $l_{2}=d_{0}+d_{1}$ is used as the input of the second iteration process, and if the number of iteration process is n, the input of the nth iteration $l_{n}=\sum_{j=1}^{n}d_{j},$ (*j* is the number of iterations). Because the mileage interval of the adjacent cross-sections obtained by the TS1 can reach 1–2 cm, the threshold of iterative convergence in the algorithm is set to 1 cm.

(1) $$\{\begin{array}{c} X_{m1}=X_{A}+l_{1}\sum_{i=1}^{4}R_{i}\cos\left[\alpha_{A}+\alpha \left(K_{A}V_{i}l_{1}+\frac{\left(K_{B}-K_{A}\right)V_{i}^{2}}{2L_{S}}l_{1}^{2}\right)\right]\\ Y_{m1}=Y_{A}+l_{1}\sum_{i=1}^{4}R_{i}\sin\left[\alpha_{A}+\alpha \left(K_{A}V_{i}l_{1}+\frac{\left(K_{B}-K_{A}\right)V_{i}^{2}}{2L_{S}}l_{1}^{2}\right)\right] \end{array}$$

(2) $$d_{1}=\left(Y_{m}-Y_{m_{1}}\right)\sin\left[\alpha_{A}+\alpha \left(K_{A}d_{0}+\frac{K_{AB}}{2L_{S}}d_{0}^{2}\right)\right]+\left(X_{m}-X_{m_{1}}\right)\cos\left[\alpha_{A}+\alpha \left(K_{A}d_{0}+\frac{K_{AB}}{2L_{S}}d_{0}^{2}\right)\right]$$

In the equations, $R_{i}$ and $V_{i}$ are constants, and the subscript i refers to the number of constants. $R_{1}=R_{4}=0.1739274226,R_{2}=R_{3}=0.3260725774$, $V_{1}=0.0694318442$, $V_{2}=0.330009478$, $V_{3}=0.6699905218$, $V_{4}=0.9305681558$. $\left(X_{A},Y_{A}\right)$ is the coordinate of starting point A of the curve unit in the route coordinate system, $K_{A}$ and $K_{B}$ are the curvatures of points A and B, respectively, $K_{AB}=K_{B}-K_{A},$ $\alpha_{A}$ is the azimuth of point *A* in the route coordinate system, $L_{S}$ is the arc length of the curve unit. α is a symbol for the trend of the curve unit; when the curve unit inclines to the right, it equals +1, and when the curve unit inclines to the left, it equals −1.

## 4. Experimental Validation and Discussion

### 4.1. Data Accquisition

The experiment was conducted in part of a Chengdu subway tunnel, which is a round shield tunnel that was recently completed and about to enter trial operation stage. The tunnel segments are spliced in the staggered joints measure, and the interior diameter of the design tunnel segment is 5400 mm. First, the mileage of the starting point was selected, and then the CNU-TS-1 tunnel monitoring trolley carried out a round trip measurement for the tunnel with a length of 100 m from the starting point to obtain the cross-sectional point cloud data at each mileage position in the tunnel. After the data acquisition process was completed, the data acquisition software was used to complete the multi-sensors data fusion and exported the point clouds in LAS format. To verify the accuracy of the system for deformation measurements, the total station instrument was used for cross-sectional measurements in the measuring area of the TS1. In addition, to ensure that the cross-sections measured by the total station are orthogonal to the tunnel, the measurement area of the total station was selected in the area that the horizontal curve line and vertical curve line of the tunnel are both the straight-line type. Finally, 19 cross-sections were measured at the interval of 2 m by the total station, and each of which includes 17 cross-sectional points.

**Figure 7:**
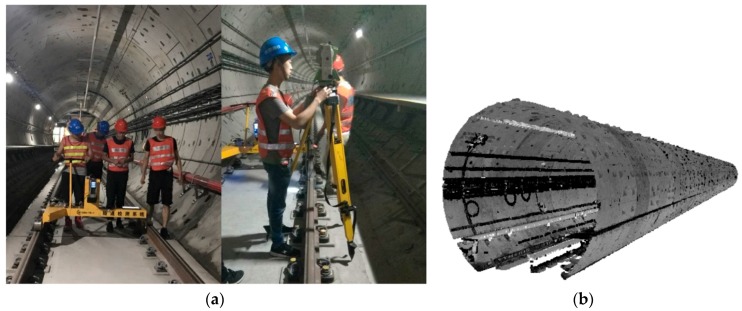
Data acquisition process. (**a**) Tunnel monitoring by the TS1 and total station; (**b**) point clouds measured by the TS1.

During the direct measurement process of the tunnel, the installation and initialization of the TS1 took approximately 10 min, the scanning of tunnel point clouds took approximately 10 min and the fusion of the multi-sensors data took approximately 25 min. Moreover, it took approximately 5 min to disassemble the equipment, and 8345 cross-sections were obtained finally. Therefore, the total time it took to measure a tunnel with a length of 100 m using the TS1 was approximately 50 min. In addition, it took approximately 15 min to measure each cross-section using the total station (including the placement of the instrument and the measurement of the sectional points). If a total station is used to measure the tunnel with a length of 100 m at the interval of 2 m, 50 cross-sections need to be obtained, and the total time is 750 (50 × 15) min; thus, the TS1 is 15 times more efficient than that of the total station and has significant advantages in the density of the cross-sections and the density of the cross-sectional points. Figure 7a shows the process of data acquisition using the TS1 and total station, and Figure 7b are the three-dimensional cross-sectional point clouds of the tunnel obtained by the TS1.

### 4.2. Accuracy Verification

**Figure 8:**
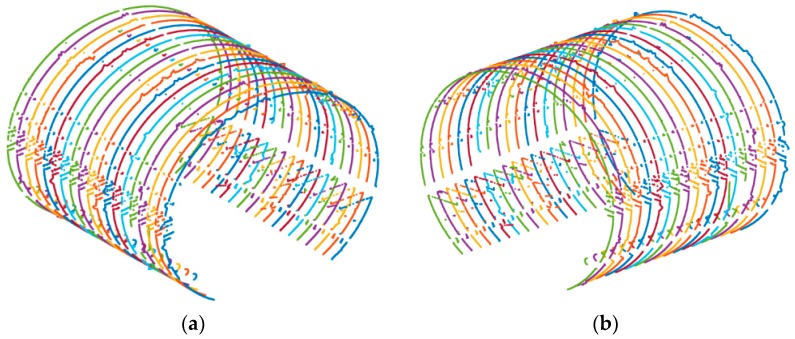
Corresponding cross-sections with total station. (**a**) Direct measurement cross-sections; (**b**) reverse measurement cross-sections.

First, the method introduced in Section 3.2 of this paper was used to calculate the mileages of the cross-sections measured by the total station. In addition, the cross-sections with the same mileages as the cross-sections measured by total station were selected from the cross-sectional point clouds obtained by direct and reverse observations through the TS1. Although the mileage interval of the cross-sections obtained by the TS1 can reach 1–2 cm, the cross-sections themselves are discrete. There is no guarantee that the cross-sectional point clouds that fully accord with the mileages of the cross-sections measured by total station exist; therefore, the nearest cross-sections to the mileages were selected. The results of selection indicate that the mileage differences of the corresponding cross-sections obtained by the TS1 and total station are all within 5 mm. Figure 8a,b is the cross-sections that were selected from the cross-sectional point clouds obtained by direct and reverse measurements, respectively, which correspond to the mileages of the cross-sections measured by total station.

#### 4.2.1. External Coincidence Accuracy Verification of the System

The convergent diameters of cross-sections measured by the total station were calculated by the method of Section 3.1.1 and used as the reference values. The noises in the cross-sectional points obtained by the TS1 were removed, and the convergent diameters of the cross-sections were calculated by the method of Section 3.1.1. The deviation of the convergent diameter between the cross-sections measured by the TS1 and total station at each mileage position was calculated and analyzed. Table 1 indicates that the deviations are between −3.1 mm and +2.2 mm, and the absolute mean values of the deviations of direct and reverse measurements are 1.6 mm and 1.4 mm, respectively. Meanwhile, the positive and negative deviation trends of the direct and reverse measurement cross-sections are consistent. Refer to the accuracy requirements for the long-term monitoring of tunnels in the “Standard for monitoring and measurement of urban rail transit structure”; the accuracy of the convergence measurement should not be less than ±3 mm. This indicates that the TS1 is suitable for tunnel deformation detection.

#### 4.2.2. Internal Coincidence Accuracy Verification of the System

**Figure 9:**
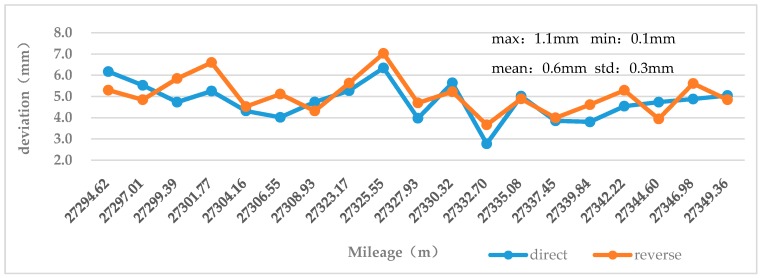
Comparison of the absolute mean deformation of points measured by round trip.

As introduced in Section 3.1.2, all the selected cross-sections were resampled and the resampling angle interval was 1°. When the resampling process was completed, the point deformation values of each cross-section were calculated, and then, the absolute mean value of the point deformation values of each cross-section was calculated. The two curved lines shown in Figure 9 illustrate the absolute mean values of point deformations of the cross-sections obtained by direct and reverse observations. As shown in the diagram, the whole deformation of each cross-section is greater than zero, and the deformation range of the cross-sections is between 3 mm and 7 mm; the average deviation of the cross-section deformation values of the direct and reverse observations is 0.6 mm; the standard deviation is 0.3 mm, all of which are within 1 mm. These indicate that although the distributions of the cross-sectional points at the same mileage position are different, there are a little effect on the results of the deformation analysis, and the point clouds obtained by the TS1 has very high stability for the tunnel deformation analysis.

#### 4.2.3. Accuracy Verification of the Simulated Deformation Method

**Figure 10:**
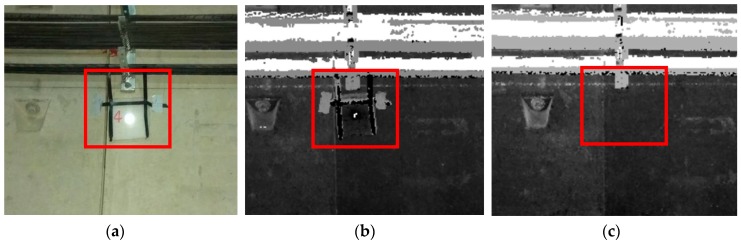
Simulation experiment. (**a**) Artificial deformation sign; (**b**) point clouds of direct measurements; (**c**) point clouds of reverse measurements.

Because the tunnel in the experimentation area had just been built, the deformations measured were very small. To further verify the accuracy, a deformation sign (a foam with a length, width of 19 cm and thickness of 1.5 cm, the reflector is pasted in the middle of the foam) was attached to the pipe joints at the waist of the tunnel’s inner wall near mileage 27,307.46 during direct measurement using the TS1. In addition, the deformation sign was removed during the reverse measurement in order to simulate the tunnel deformation to verify the measurement accuracy, which is shown in Figure 10.

**Figure 11:**
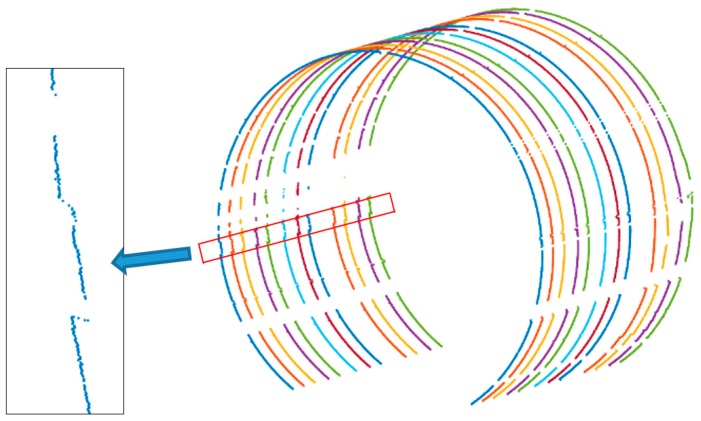
Cross-sectional points of the simulation experimental zone (after denoising).

Due to the change of force, the deformation of the tunnel is generally distributed in face pattern rather than a point pattern. Therefore, the actual deformation area is considered to be the area where the continuous deformation occurs at a certain angle interval in the same cross-section; otherwise, it is considered a local effect due to ineffective noise removal. In the experimental area where the deformation sign was located, 10 cross-sections were evenly selected from the point cloud data obtained by direct and reverse measurements respectively, and the deformation analysis was carried out. The cross-sections obtained by the direct measurement indicate that each cross-section has an obvious continuous negative deformation between 187° and 191°. Using the angle difference (4°) and theoretical radius (2.7 m), the arc length can be calculated as 18.8 cm, which basically coincides with the actual length of the deformation sign. Figure 11 is the overall view of the cross-sections obtained by direct measurements in the artificial deformation area and the local view of the point clouds of the deformation sign.

The mean deformation between the angle intervals of 187° and 191° of each cross-section is calculated, which is shown in Table 2. The results indicate that the deformations from the fourth to sixth cross-sections in the table are larger, mainly due to the thickness of the reflector in the middle of the deformation sign. Meanwhile, the average deviation of cross-sections measured by direct and reverse measurements in the deformation area is 16.1 mm, and the thickness of the artificial deformation sign is 15 mm, indicating that the accuracy of the system for the tunnel measurement is within 1.1 mm. Furthermore, as presented in the previous part of the article, the measurement accuracy of laser scanner is ±2 mm, which is close to the measurement accuracy of the TS1. This proves the applicability of the TS1 for tunnel deformation detection.

## 5. Discussion

Although the TS1 has achieved high accuracy in deformation monitoring, there are still some problems that must be solved. First, the system is only suitable for the tunnel with a track that has a standard gauge of 1435 mm and is not applicable to the monitoring of other types of tunnels. In addition, the way in which measurements are carried out through human push, the uneven walking speed leads to an uneven density of cross-sectional point clouds. In the later period, we will study the second generation of the TS1, which uses an electric automation design and the modular processing of the measurement system to adapt to different tunnel or pipeline measurements. Second, our system uses odometers to locate the cross-sections. Although the mileage data obtained by the odometers have been strictly corrected, the location accuracy still needs to be improved. Furthermore, we only verified the accuracy of deformation detection based on the original data in the vehicle body system in this paper, not the absolute accuracy of the system for point location. This mainly because, for the tunnel in operation stage, the maintenance workers mostly rely on the ring number of the tunnel wall or a certain mileage reference position to locate the deformation, and the absolute position of the tunnel is not very useful. The practical significance of the absolute position of tunnel measurements at the operation stage is still debated, and the main purpose of the system is to analyze the relative deformation of the tunnel according to the actual measured data. In the future, we will study the algorithms to transform the dynamic two-dimensional cross-sections into the absolute coordinate system. Thus, the true line type of the tunnel can be restored, and more abundant data can be provided to support the tunnel detection.

## 6. Conclusions

Based on the three-dimensional laser scanning technology, the CNU-TS-1 mobile tunnel monitoring system was developed and introduced in this paper, which includes a tunnel monitoring trolley, data acquisition software and data processing software. In the process of data acquisition, the system uses odometers to locate the cross-sections and then corrects the data through the longitudinal joints of the tunnel segment lining and the mileages of the starting point and end point. This method not only significantly reduces the cost of the system but can also accurately locate the deformed area. Meanwhile, it can efficiently obtain the cross-sections that are orthogonal to the tunnel in a dynamic way and analyze the relative deformation of the tunnel by the measured data. The whole process does not need a control measurement, which eliminates the dependence on tunnel design data in traditional methods. In addition, through the data processing software of the system, the deformation of any selected cross-section can be analyzed, and the point deformation values at all angles can be obtained and quickly exported in the form of the analysis report; thus, the hidden danger in the tunnel can be detected quickly and positioned accurately, and a timely warning can be provided. Experiment results in the Chengdu subway tunnel indicate that the density of the adjacent cross-sections and the density of the cross-sectional points obtained by TS1 have been greatly improved, which can provide a basis for the detailed analysis of tunnel deformation. Furthermore, the accuracy of cross-section measurement of the system can reach 1.1 mm, which is close to the accuracy of the laser scanner. In addition, TS1 is 15 times more efficient than that of the total station. These results demonstrate the applicability of the system for tunnel deformation detection and its advantages in efficiency and point density compared with the traditional method.

## Figures and Tables

**Table 1:** Convergent diameter deviation of cross-sections measured by TS1 and total station.

Mileage	Total Station (m)	Direct (m)	Reverse (m)	Deviation of Direct (mm)	Deviation of Reverse (mm)
27,294.62	2.7061	2.7063	2.7063	0.2	0.2
27,297.01	2.7073	2.7060	2.7050	−1.3	−2.3
27,299.39	2.7070	2.7045	2.7057	−2.5	−1.4
27,301.77	2.7071	2.7056	2.7050	−1.5	−2.0
27,304.16	2.7050	2.7046	2.7040	−0.4	−1.0
27,306.55	2.7061	2.7035	2.7043	−2.6	−1.7
27,308.93	2.7046	2.7052	2.7049	0.6	0.3
27,323.17	2.7051	2.7050	2.7051	−0.1	0.0
27,325.55	2.7069	2.7055	2.7050	−1.4	−1.9
27,327.93	2.7058	2.7038	2.7045	−2.0	−1.3
27,330.32	2.7047	2.7059	2.7053	1.2	0.6
27,332.70	2.7058	2.7027	2.7038	−3.1	−2.0
27,335.08	2.7064	2.7050	2.7051	−1.4	−1.3
27,337.45	2.7054	2.7040	2.7040	−1.5	−1.4
27,339.84	2.7065	2.7037	2.7047	−2.8	−1.7
27,342.22	2.7065	2.7048	2.7055	−1.7	−1.0
27,344.60	2.7074	2.7051	2.7042	−2.2	−3.1
27,346.98	2.7069	2.7050	2.7059	−1.9	−1.0
27,349.36	2.7031	2.7053	2.7050	2.2	1.9
Absolute mean				1.6	1.4

**Table 2:** Comparison of deformation values in the simulated experiment area.

Cross-Section	1	2	3	4	5	6	7	8	9	10	Mean
Direct	−10.4	−12.2	−12.7	−14.0	−13.4	−14.3	−11.1	−10.2	−8.4	−8.7	−11.5
Reverse	5.5	3.4	3.8	4.6	5.8	4.5	3.9	4.1	5.2	5.3	4.6
Deviation	16.0	15.6	16.5	18.5	19.2	18.7	15.0	14.2	13.5	14.1	16.1
